# Sudden Infant Death Syndrome and Safe Sleep on Twitter: Analysis of Influences and Themes to Guide Health Promotion Efforts

**DOI:** 10.2196/10435

**Published:** 2018-09-07

**Authors:** Kelly A Pretorius, Michael Mackert, Gary B Wilcox

**Affiliations:** 1 School of Nursing The University of Texas at Austin Austin, TX United States; 2 Center for Health Communication Moody College of Communication and Dell Medical School The University of Texas at Austin Austin, TX United States; 3 Stan Richards School of Advertising and Public Relations The University of Texas at Austin Austin, TX United States; 4 Department of Population Health The University of Texas at Austin Austin, TX United States; 5 Department of Statistics and Data Sciences The University of Texas at Austin Austin, TX United States

**Keywords:** sudden infant death, sudden unexpected infant death, accidental suffocation in a sleeping environment, infant mortality, safe sleep, sleep environment, social media, Twitter, health communication, public health

## Abstract

**Background:**

In the United States, sudden infant death syndrome (SIDS) is the leading cause of death in infants aged 1 month to 1 year. Approximately 3500 infants die from SIDS and sleep-related reasons on a yearly basis. Unintentional sleep-related deaths and bed sharing, a known risk factor for SIDS, are on the rise. Furthermore, ethnic disparities exist among those most affected by SIDS. Despite public health campaigns, infant mortality persists. Given the popularity of social media, understanding social media conversations around SIDS and safe sleep may assist the medical and public health communities with information needed to spread, reinforce, or counteract false information regarding SIDS and safe sleep.

**Objective:**

The objective of our study was to investigate the social media conversation around SIDS and safe sleep to understand the possible influences and guide health promotion efforts and public health research as well as enable health professionals to engage in directed communication regarding this topic.

**Methods:**

We used textual analytics to identify topics and extract meanings contained in unstructured textual data. Twitter messages were captured during September, October, and November in 2017. Tweets and retweets were collected using NUVI software in conjunction with Twitter’s search API using the keywords: “sids,” “infant death syndrome,” “sudden infant death syndrome,” and “safe sleep.” This returned a total of 41,358 messages, which were analyzed using text mining and social media monitoring software.

**Results:**

Multiple themes were identified, including recommendations for safe sleep to prevent SIDS, safe sleep devices, the potential causes of SIDS, and how breastfeeding reduces SIDS. Compared with September and November, more personal and specific stories of infant loss were demonstrated in October (Pregnancy and Infant Loss Awareness Month). The top influencers were news organizations, universities, and health-related organizations.

**Conclusions:**

We identified valuable topics discussed and shared on Twitter regarding SIDS and safe sleep. The study results highlight the contradicting information a subset of the population is exposed to regarding SIDS and the continued controversy over vaccines. In addition, this analysis emphasizes the lack of public health organizations’ presence on Twitter compared with the influence of universities and news media organizations. The results also demonstrate the prevalence of safe sleep products that are embedded in safe sleep messaging. These findings can assist providers in speaking about relevant topics when engaging in conversations about the prevention of SIDS and the promotion of safe sleep. Furthermore, public health agencies and advocates should utilize social media and Twitter to better communicate accurate health information as well as continue to combat the spread of false information.

## Introduction

Sudden infant death syndrome (SIDS) is the leading cause of death in infants aged between 1 month and 1 year and the third leading cause of infant mortality in the United States [1]. Approximately 3500 infants die every year due to SIDS and sleep-related reasons [2]. Causes of sleep-related deaths include SIDS, accidental suffocation and strangulation in bed (ASSB), and ill-defined deaths [2].

While there was a marked decline in sleep-related deaths following the American Academy of Pediatrics (AAP) recommendations for supine sleep position in 1994, this decline has since lost momentum [2-4]. Furthermore, there appears to be an increase in the number of unintentional sleep-related deaths in infants. For example, infant mortality rates due to ASSB have quadrupled since 1984 [5]. While bed sharing is a known risk factor for infant death [6], bed sharing has increased in the United States, with 50%-61% of mothers admitting to bed sharing at some point [6,7] and African American women specifically bed sharing with infants for perceived safety, improved infant sleep, and convenience [4,8].

To make matters more complex, sociocultural differences among sleep behaviors and mortality rates persist. For instance, populations at the highest risk for infant mortality due to SIDS and sleep-related deaths exhibit the following characteristics: lower socioeconomic status, uneducated, younger age of birth mother, smoking during pregnancy, and African American or American Indian or Alaskan ethnicity [1,9,10].

In 2016, the AAP updated their recommendations for the prevention of SIDS and sleep-related infant deaths [2]. However, despite ongoing communication efforts, there appears to be a gap between public health recommendations to prevent SIDS and promote safe sleeping environments and what parents are actually practicing with regard to infant sleeping environments, as evidenced by the continued infant mortality statistics [1]. Media coverage of recommendations, as well as social media conversations around SIDS and safe sleep, has the potential to spread, reinforce, or counteract public health recommendations.

Analyzing social media, an incredibly popular and useful tool with wide-reaching capabilities, allows for a better understanding of some beliefs and exposure to information regarding SIDS and may, therefore, assist in the development of more effective public health programs. As of 2015, approximately 65% of American adults use social media [11]. A recent trend reflects that people are also using social media for health information. According to the Health Information National Trends Survey (HINTS), a survey that tracks health communication and information technology of American adults, social media was found to penetrate all persons despite race, education, or health care access [12]. This finding is also supported by studies demonstrating the widespread use of social media among parental groups [13-18].

Moreover, Twitter is gaining popularity and is used by 24% of American adults [19]. The average Twitter user tends to be younger (36% of those aged between 18 and 29 years use Twitter) [19]. Twitter use spans educational levels, although those with college degrees use it more than those with a high school degree or less (29% vs 20%) [19]. In one study of primarily female (76%), African American mothers (41%), 12.5% participants used Twitter daily [20]. Understanding Twitter’s users is essential when considering the health issue of SIDS, given that SIDS impacts African American people disproportionately more than other ethnicities [1,2,9].

Therefore, there is an opportunity to use social media platforms, like Twitter, for listening to subgroups of the public, as well as for health promotion. In addition, Twitter can be used to assess the public’s response and shape the public’s perception of a health issue, thereby allowing health communicators to more effectively plan and implement response strategies [21,22]. Moreover, Twitter can assist policy makers and governmental agencies in understanding the information that is being spread and in potentially combating the spread of false information [23]. Furthermore, Twitter is an ideal form of social media for analysis given that the posts are public.

The purpose of our study was to better understand social media conversation around SIDS to understand possible influences and guide health promotion efforts. The remainder of this paper outlines the specific study methods, findings, and implications of this work for public health research and practice.

## Methods

### Text Analytics and Data Acquisition

We used textual analytics to identify topics and extract meanings contained in unstructured textual data. Twitter messages were captured for 3 months—September, October, and November 2017. Tweets and retweets were collected using NUVI software (NUVI, Lehi, UT, USA) in conjunction with Twitter’s search API (application programming interface). Because our focus was on SIDS, keyword combinations were selected to avoid biased language used by different audiences, filter irrelevant information, and increase the likelihood of capturing a greater signal-to-noise ratio for the topic under investigation. We used the following keywords or phrases to capture relevant Twitter messages: “sids,” “infant death syndrome,” “sudden infant death syndrome,” and “safe sleep.”

The keywords returned a total of 41,358 messages. There were 5282 messages in September 2017, 13,438 in October, and 22,638 in November. Then, we analyzed the messages using text mining and social media monitoring software, SAS Text Miner 12.1 (SAS Institute Inc, Cary, NC, USA) [24] and NUVI [25], and interpreted the findings. NUVI is a social listening tool that allows for the monitoring of messages from Twitter using basic aggregation tools based mainly on the frequency and reach of users, posts, and keywords. Using NUVI, the tweets were analyzed for trending hashtags, top influencers (determined primarily by the number of followers and frequency of mentions and retweets), and location of tweets by state and per capita (analyzed using a combination of geo-tagging and specific locations associated with the Tweeter’s bio). The use of publicly available data in this study did not require approval from our Institutional Review Board.

### Text Analytics

The tweets’ textual content was analyzed using SAS Text Miner 12.1 [24]. SAS Text Miner is an algorithm-driven statistical software used to uncover and understand information. SAS Text Miner provides the ability to parse and extract information from text, filter and store the information, and assemble tweets into related topics for introspection and insights from the unstructured data [26,27].

The first step was to extract, clean, and create a dictionary of words using a natural language processor. Using a Text Parsing node, each message was divided into individual words; these words were listed in a frequency matrix, and words that contributed little to the understanding of the topic, such as auxiliary verbs, conjunctions, determiners, interjections, participles, prepositions, and pronouns, were excluded from the analysis. Following this, a Text Filter node was used to exclude words that appeared in <4 messages as a conservative measure to reduce noise. A single author with the knowledge of the subject matter visually inspected and manually removed irrelevant terms. The words initially included (and excluded) in the analysis were visually inspected to ensure accuracy and identify unrecognizable symbols and letter groups for exclusion.

With the inclusion criteria set, a Text Topic node was used to combine terms into 8-12 topic groups. This clustering divided the document collection into mutually exclusive groups based on the presence of similar themes using expectation maximization clustering. After visually examining each of the created topics, 10-, 11-, and 8-topic solutions most clearly illustrated the main themes for September, October, and November, respectively. Finally, the researchers reviewed the individual messages of the final topic groups to interpret the final themes. This was accomplished by individually reviewing the actual messages from each cluster or topic to arrive at the description that is now contained in the tables identifying the themes.

## Results

### Themes

Data on SIDS and safe sleep tweets for the months of September, October, and November 2017 have been presented in Tables 1-3, respectively.

A major theme identified was safe sleep recommendations. In September and October, 3 of the topics included recommendations for safe sleep to prevent SIDS, including the updated AAP recommendations. In November, just 2 of the topics included recommendations for safe sleep to prevent SIDS, including the AAP recommendations. In September, some of these messages originated from public health agencies, including a video from the National Institutes of Health promoting safe sleep, and statewide efforts to reduce SIDS. This was slightly different from October, where a popular story originated from CNN and included advertising for sleep products, such as Dr Harvey Karp’s responsive bassinet, Snoo. In November, the safe sleep messaging originated from public health agencies, including statewide efforts to reduce SIDS; however, some of them were also personal recommendations on infant sleep and SIDS prevention.

**Table 1:** Sudden infant death syndrome (SIDS) and safe sleep tweets by topic for September 2017.

Topic	n (%)	Description
+sleep,+safe,safe sleep,baby,+share	459 (8.69)	Safe sleep recommendations to reduce SIDS (some originating from public health agencies, some about recent studies on SIDS)
sudden,syndrome,sudden infant death syndrome,+death,+infant	428 (8.10)	Explanations for SIDS (vaccines cause Autism, brainstem abnormality causes SIDS, cardiac-mediated SIDS), Web-based community fundraising for SIDS, baby box initiatives, and the American Academy of Pediatrics (AAP) guidelines on safe sleep
Institute,+american,support,charity,+cause	219 (4.15)	Web-based fundraising for the American SIDS Institute
Research,+confirm,+brain,chemistry,babies’ brain	212 (4.01)	Australian study links brain chemical to SIDS
undesa,political leadership,parisagreement,cop,political	112 (2.12)	AAP recommendations for safe sleep and unrelated posts regarding small island development
doritmi,emmagpaley,nicolasdenver,badzoot7,markjarthur	104 (1.97)	Explanations for SIDS (vaccines cause SIDS, vaccines do not cause SIDS) and daycare complies with safe sleep guidelines
Grateful https,penny,funeral,grateful,217xxmvzmk	88 (1.67)	Fundraising for SIDS and general information about SIDS
Huffpost,easy,a-alone,b-back,c-crib	87 1.65)	Safe sleep recommendations to reduce SIDS
+back,williamdevry1,nancyleegrahn,julexis,gh	72 (1.36)	Explanations for SIDS (vaccines cause SIDS)
maine,vaccine,mcvc,dhhs,+notify	68 (1.29)	Explanations for SIDS (vaccines cause SIDS)

**Table 2:** Sudden infant death syndrome (SIDS) and safe sleep tweets by topic for October 2017.

Topic	n (%)	Description
+baby,+sleep,+sleep,+child,+safe	1549 (11.53)	Safe sleep recommendations to reduce SIDS, personal story of infant lost to SIDS, and advertising for a safe sleep product (Baby Box University)
+game,nfl,+player,+time,+season	1188 (8.84)	Football discussion that included information on daughter of a National Football League football player who died from SIDS
+risk,breastfeeding,+researcher,+study,+month	1004 (7.47)	Breastfeeding reduces SIDS (recent study on SIDS)
aidan,+loss,+cemetery,+child,remains	517 (3.85)	October as National Pregnancy and Infant Loss Awareness Month (personal stories of pregnancy and infant loss due to SIDS)
+swaddle,+sleep,+myth,unexplained,+sleep	488 (3.63)	Safe sleep recommendations to reduce SIDS and advertising for sleep products (Dr Harvey Karp’s bed, baby hammock)
+baby,+pacifier,baby,mortality,+bed	337 (2.51)	Safe sleep recommendations to reduce SIDS and advertising for Baby Box University
+substance,+brain,+abnormality,+river,Adelaide	329 (2.45)	Australian study links brain chemicals to SIDS
finnbin,smartmom,safe,sleep,+school	315 (2.34)	Finnbin Box partners with Web-based mom group to increase safe sleep, and advertising for baby box, SafeSleepSchool.com
mata,+adoption,eac,violah,davises	277 (2.06)	Personal story of infant loss due to SIDS
drpaolini,and_kell,steffieschiltz,lilearthling369,markjarthur	241 (1.79)	United States and vaccines cause SIDS
+day care,broadway,+care,oregon,+facility	228 (1.70)	Baby dies from SIDS in Oregon at daycare

**Table 3:** Sudden infant death syndrome (SIDS) and safe sleep tweets by topic for November 2017.

Topic	n (%)	Description
â,+baby,+child,+sleep,+sleep	3063 (13.53)	Safe sleep recommendations to reduce SIDS, vaccines cause SIDS, advertising for infant sleep products (book on sleep, Sleep N Feed), and baby box initiatives (in Tennessee, Minnesota, and Colorado)
+vaccine,+cause,autism,+cancer,myonesciencetweet	1630 (7.20)	Explanation of SIDS (vaccines do not cause SIDS)
+smoke,tobacco,+smoke,secondhand,secondhand smoke	1595 (7.05)	Antitobacco information (includes statement that smoking increases SIDS)
breastfeeding,+study,+risk,+month,+researcher	1495 (6.60)	Breastfeeding reduces SIDS (recent study on SIDS)
gaither,+alcohol,+drug,+pregnancy,+birth	680 (3.00)	Risks of smoking and alcohol while pregnant (including risk of SIDS), Muslim habits versus that of the West (regarding behavior while pregnant and risk of SIDS), and high infant mortality rates in the United States (possibly due to SIDS)
step2,halo,snoozypod,carle,+giveaway	623 (2.75)	Halo SleepSack information, including advertisement and Web-based giveaway (founders lost infant to SIDS), personal recommendations on sleep, and how to prevent SIDS
+thermometer,shu,avery,+monitor,+technology	395 (1.74)	Report on health-monitoring technologies for babies to prevent SIDS
and_kell,kenjaques,vbalance03,badzoot7,drpaolini	224 (0.99)	Explanation for SIDS (vaccines do not cause SIDS)

Another theme identified was safe sleep devices. In November, safe sleep messaging included advertising for the following: Baby Box Co, Baby Box University, and a baby hammock. The concept of a baby box was also present in October, where 3 topics specifically discussed the concept of a baby box, Baby Box University, and Finnbin Box. In November, 2 topics were related to safe sleep devices; one topic included baby box initiatives, and advertising for the Halo SleepSack was also found. In November, an entire topic was dedicated to infant health-monitoring devices and SIDS.

A third theme identified was the potential cause of SIDS. In September, 5 of the topics were related to the causes of SIDS and identified the following as contributing to SIDS: vaccines, alteration in serotonin, and brainstem abnormality or cardiac-mediated SIDS. Within this category, however, it was also mentioned that vaccines do not cause SIDS. In October, 2 themes were related to the potential cause of SIDS: one was tweets calling out individuals and claiming that the United States and vaccines cause SIDS; the other was about an Australian study that found alterations in serotonin contributes to SIDS. Finally, in November, 2 of the topics included information on the cause of SIDS, including a transcript from an antivaccine conference where vaccines are claimed to cause SIDS, as well as a tweet stating vaccines do not cause SIDS.

The fourth theme identified was a recent study’s findings that breastfeeding reduces SIDS. Additionally, the risk associated with smoking and alcohol use during pregnancy was a theme. Within this topic was a detailed comparison of Muslim habits versus those of the West with regards to smoking and alcohol use during pregnancy.

Finally, the month of October demonstrated 5 topics that detailed personal and specific stories of infant loss. These stories included those of a football player who lost an infant and a baby that died in an Oregon daycare from SIDS. This was expected as October is Pregnancy and Infant Loss Awareness Month [28].

### Influencers and Reach

Data on top influencers for SIDS and safe sleep for the months of September, October, and November 2017 have presented in Tables 4-6, respectively.

The top influences were news organizations, universities, and health-related organizations, such as the Centers for Disease Control and Prevention and WebMD. One top influencer, The Australian, shared information about an Australian study linking brain chemicals to SIDS; this study’s findings continued in its popularity throughout this analysis.

Another top influencer, ABS-CBN News, a Filipino news organization, posted about breastfeeding and reducing the risk of SIDS, while another top influencer, Forbes Health, later posted about formula not doubling the risk of SIDS. Likewise, other top influencers, WebMD, Today’s Parent, and Norton Healthcare, posted about how breastfeeding reduced the risk of SIDS.

Thus, the topics posted by the top influencers primarily addressed the Australian research study linking brain chemicals to SIDS, risk factors for SIDS (alcohol use), and SIDS prevention (primarily discussing the importance of breastfeeding).

**Table 4:** Sudden infant death syndrome (SIDS) and safe sleep top influencers for September 2017.

Influencer	Posted description of the influencer	Number of followers	Post topic
The Australian, @australian	News from The Australian newspaper and The Australian Online.	644,471	Australian study linking brain chemicals to SIDS
United Nations Human Rights, @UNHumanRights	The United Nations #HumanRights office is led by High Commissioner #Zeid.	1,834,760	Unrelated topic, standing up for human rights
Uni of Adelaide, @UniofAdelaide	Official account with answers for your uni questions.	43,925	Australian study linking brain chemicals to SIDS
CDC_NCBDDD, @CDC_NCBDDD	CDC’s Center protecting those most vulnerable to health risks: babies, children, people with blood disorders, and people living with disabilities.	6408	Alcohol use during pregnancy can also lead to miscarriage, stillbirth, and SIDS

**Table 5:** Sudden infant death syndrome (SIDS) and safe sleep top influencers for October 2017.

Influencer	Posted description of the influencer	Number of followers	Post topic
ABS-CBN News, @ABSCBNNews	Stories, video, and multimedia for Filipinos worldwide, from ABS-CBN News and Current Affairs, the Philippines’ most trusted news organization.	5,588,990	Breastfeeding lowers risk of SIDS
Rutgers University, @RutgersU	Rutgers, The State University of New Jersey, is a leading public research university.	124,465	SIDS prevention
Tesco, @Tesco	Every little helps. Follow us for quick and easy recipes, food inspiration and helpful home hacks.	512,320	Unrelated topic (insect is pronounced “soh-sids”)
The Advertiser, @theTiser	Breaking news and features from The Advertiser and Sunday Mail.	131,246	Australian study linking brain chemicals to SIDS

**Table 6:** Sudden infant death syndrome (SIDS) and safe sleep top influencers for November 2017.

Influencer	Posted description of the influencer	Number of followers	Post topic
Forbes Health, @ forbeshealth	@Forbes news covering the business of big pharma, health care and science.	74,653	Formula feeding does not double risk of SIDS
WebMD, @WebMD	WebMD and our medical team bring you the most trust-worthy and timely health news and information.	2,576,232	Study supporting breastfeeding reducing risk of SIDS
Today’s Parent, @Todaysparent	“The days are long, but the years are short.” Canada’s #1 parenting brand.	2,526,850	Study supporting breastfeeding reducing risk of SIDS
Norton Healthcare, @Norton_Health	Official page. Caring for our community and tweeting because we believe #health should keep up life.	6390	Study supporting breastfeeding reducing risk of SIDS

**Table 7:** Trending hashtags (by order of occurrence and month).

Month	Trending hashtags
September	#sids, #vaccines, #unga, #hr36, #safesleep365, #autism, #vaxxed
October	#sids, #vaccines, #safesleep, #sidsawarenessmonth, #caboverde, #learntherisk, #uxnow
November	#myonesciencetweet, #thankyoursid, #sids, #cop23, #nottobacco, #280characters, #vaccines

The organizations with the most reach varied by month and included goodhealth, ABS-CBN News, and WebMD. These organizations shared the following messages: swaddling may put babies at risk for SIDS and breastfeeding reduces SIDS. Notably, there is no dominant social media voice related to this issue, with reach driven primarily by topical news stories.

### Hashtags and Most Shared Mentions

The trending hashtags related to the topics have been presented in Table 7.

When comparing the trending hashtags, the topic of vaccines persisted throughout September, October, and November. For instance, “#vaccines” was the second most common hashtag in September and October. “#autism” came in as the sixth most common hashtag in September; this highlights the continued debate among the public regarding the possible link between vaccines and SIDS, as well as autism, despite public health efforts.

Furthermore, a marked variation existed among the most shared positive mentions, identifying the popularity of stories of personal loss as well as efforts of the AAP to educate the public on safe sleep and SIDS. In September, the most shared positive mention was a tweet from a host on NBC Sports with 25,367 followers, asking for donations to a friend who lost their 4-month old to SIDS. In October, the most shared positive mention was from HealthyChildren, the official parenting website of the AAP, with 39,082 followers, offering information about placing babies to sleep on their back as well information about SIDS and safe sleep environments. It is worth highlighting that this is the only mention of a safe sleep environment when reviewing the top influencers, those with the most reach, and the most shared positive mentions. The most shared negative mention of the keywords occurred in November, when Doc Bastard, with 16,508 followers tweeted *“Vaccines do not cause autism, SIDS, autoimmune disorders, diabetes, or cancer. They cause adults.”*

## Discussion

### Principal Findings

Analyzing social media to better understand a subset of the population’s perceptions and exposure to information is a helpful and essential tool. This analysis of conversations surrounding the concepts of SIDS and safe sleep has helped identify major themes that otherwise would have persisted without acknowledgment from the medical or public health communities. In understanding the topics and themes that are popular on social media, health care and public health professionals can begin to address some of the common misconceptions and beliefs regarding SIDS and safe sleep. Furthermore, this analysis has been helpful in pointing out the general lack of public health agencies’ presence on social media. With the information gained from this analysis, health care professionals and those in the public health arena can begin to have a more directed dialogue with the public and engage in more effective communication about this topic with the goal of ultimately reducing the incidence of SIDS due to unsafe sleep environments.

There is an opportunity for health care providers to engage in conversation with families about conflicting information they may be exposed to via social media, with the goal of providing accurate information. In this analysis, we identified themes that demonstrated conflicting statements that may be confusing for the average social media user. For instance, there is the mixed message of vaccines causing SIDS and vaccines not causing SIDS; this conflicting and contradicting finding is similar to what was found in an analysis of Twitter to better understand emerging topics and current vaccine perspectives [29]. In that analysis of Twitter data, there was an identified prevalence of negative sentiment surrounding vaccines as well as distrust toward organizations that deliver scientific evidence. Furthermore, the general conflicting ideas among themes are consistent with the identification of confusion and misunderstanding that was found in a study attempting to better understand the misunderstanding regarding and misuse of antibiotics through a Twitter analysis [22]. Therefore, this identified theme provides an opportunity for health care and public health professionals to openly address this confusion and reassure families that vaccines do not cause SIDS.

Another area of opportunity was highlighted in October. There was markedly high Twitter activity in October, which was expected given that October was Pregnancy and Infant Loss Awareness Month [28]. However, this appeared to lend itself to more stories of personal loss rather than an increased public health presence to promote safe sleep awareness or SIDS prevention. Similarly, another study found that tragic deaths were highlighted rather than AAP guidelines for safe sleep reviewed in an analysis of websites to determine the accuracy of safe sleep [30]. Thus, health care professionals and public health agencies should respond by pairing personal stories of loss with recommendations for safe sleep and SIDS prevention. Because in October, there was an increase in the overall activity, but not specifically regarding safe sleep and SIDS prevention, this creates an opportunity for continued education and discussion of safe sleep recommendations originating from either health care professionals or public health agencies. As an example of outreach, researchers working in this area could coordinate with institutional communication offices to share and spread their work; such an approach is likely to have a substantial reach given the power of institutional press releases and communication channels in this analysis.

In addition, health care providers and public health agencies should address the amount of advertising noted in this analysis. For instance, much of the safe sleep recommendations and guidelines were embedded with advertising for safe sleep products. Some of these products follow and promote AAP guidelines for safe sleep, while others, such as the baby hammock, do not. The popularity of baby box initiatives and infant health-monitoring devices should also be recognized. This finding of incomplete congruence of safe sleep recommendations among advertised products is similar to prior findings of inaccurate safe sleep portrayed online [30]. Understanding the commonality of advertised safe sleep devices and infant health-monitoring devices is important so that practitioners can engage in more productive and effective communication with patients regarding the topic of SIDS and safe sleep behavior. Furthermore, public health agencies can address this issue by openly commenting on the lack of evidence to support many of these products, as well as the potential hazards associated with them.

Finally, public health campaigns and efforts should utilize university and news outlets to reach out to public for educational and more effective health communication regarding the topic of SIDS and safe sleep recommendations; this statement is based on the finding that the major influencers were noted to be universities and news media organizations. Furthermore, as previously mentioned, it is emphasized that public health organizations need to play a more active role in combating false information that is so readily shared via social media outlets; this sentiment is also shared by other researchers who have analyzed social media [21,22,31].

### Limitations

When considering the implications of this work and directions for future research, it is necessary to recognize this study’s limitations. Although the Twitter users have been profiled [19], the findings cannot be completely generalized to the American population. Therefore, the themes identified may only apply to a subset of the American population. Additionally, the validity of the postings on social media may not have been intended to be taken at face value and may include gross exaggerations. Thus, generalizing from these statements may not be a completely accurate reflection. Furthermore, top influencers were determined by the number of followers, mentions, and shares rather than the number of retweets. A different algorithm could potentially identify other top influencers and, thus, impact the findings of this analysis. Moreover, the time period during which the analysis was performed could impact the identified topics. Efforts were made to include September, October, and November to adequately capture the potential effects of October being Pregnancy and Infant Loss Awareness Month. However, a different time period may have resulted in different topics surrounding SIDS and safe sleep. Furthermore, analyzing 3 consecutive months does not control for carryover effect and may have impacted the identified monthly themes.

### Conclusions

This study offers valuable information regarding what a subset of the American population believes and is exposed to with regards to SIDS and safe sleep. It highlights the contradicting information that the public is exposed to regarding SIDS and the continued controversy over vaccines. Furthermore, this analysis emphasizes the lack of public health organizations’ presence on Twitter compared with the influence of universities and news media organizations. This study also demonstrates the prevalence of safe sleep products that are embedded in safe sleep messaging. Such findings can assist health care providers in speaking about relevant topics when engaging in conversations about the prevention of SIDS and promotion of safe sleep. Public health agencies and advocates should further utilize social media and Twitter to better communicate accurate health information as well as continue to combat the spread of false information.

## Abbreviations

AAP: American Academy of Pediatrics
API: application programming interface
ASSB: accidental suffocation and strangulation in bed
HINTS: Health Information National Trends Survey
SIDS: sudden infant death syndrome
